# Virtual support for paediatric HIV treatment decision making

**DOI:** 10.1136/archdischild-2014-307019

**Published:** 2014-12-30

**Authors:** Kirsty Le Doare, N E Mackie, S Kaye, A Bamford, S Walters, C Foster

**Affiliations:** 1 St. Mary's Hospital, Imperial College NHS Trust, London, UK; 2 Department of Paediatrics, Imperial College London, London, UK; 3 Great Ormond Street Hospital, London, UK

**Keywords:** HIV, Information Technology, Pharmacology

## Abstract

**Objective:**

The objective of this study is to review clinical outcomes of recommendations made by a multidisciplinary paediatric virtual clinic (PVC) for complex case management of paediatric HIV as a model of care within a tertiary network.

**Design:**

A retrospective review of the clinical outcomes of paediatric and adolescent (0–21 years) referrals to the PVC at St. Mary's Hospital, Imperial College Healthcare NHS Trust, London was performed between October 2009 and November 2013.

**Results:**

234 referrals were made for 182 children from 37 centres, discussed in 42 meetings (median age 13 years, IQR 10–15 years). Reasons for referral included virological failure (44%), simplification of the current regimen (24%) and antiretroviral drug complications (24%). At latest follow-up, PVC advice had been instituted in 80% of referrals. Suppression following virological failure was achieved in 48% following first referral and 57% following subsequent discussions and was maintained in 95% of children referred for regimen simplification. Following advice, dyslipidaemia resolved in 42% and liver function normalised in 73% with biochemical hepatitis. Adherence support aided resolution of viraemia in nine children and 12% of referrals resulted in additional support, including psychology, social services and mental health input.

**Conclusions:**

Combined multidisciplinary virtual input with adult expertise in resistance and newer agents, paediatric knowledge of pill swallowing, childhood formulations/weight banding and parental support, assists complex treatment decision making in paediatric HIV infection. The Virtual Clinic model could be applied to the management of other rare complex diseases of childhood within a clinical network.

**What is already known on this topic**

- A perinatally HIV-infected child now has a chronic disease rather than a progressive, fatal one.
- Challenges for clinicians include selection of optimal antiretroviral drug regimen with limited paediatric formulations, pharmacokinetic, safety and efficacy data and extensive drug resistance.
- Low numbers of children living with HIV in the UK limits the treatment experience of individual clinicians.

**What this study adds**

- The virtual clinic illustrates a useful model of care for rare chronic diseases in paediatric networks.
- Combining adult and paediatric expertise enables complex decision making including access to treatment options, for example, newer off-label agents.

## Introduction

Perinatally infected infants and children are now surviving to adulthood with life-long HIV infection and long-term exposure to highly active antiretroviral therapy (HAART). To standardise and ensure optimal care across the UK for the 1131 children currently in active follow-up in the UK and Ireland,^1^ the Children's HIV Association (CHIVA) has developed standards for paediatric HIV. These emphasise the development of clinical networks with larger, tertiary centres linked to smaller paediatric services.^2^

The London HIV Consortium Paediatric Sub-Group established networks for the care of children infected with HIV in 2004, followed by the setting up of paediatric HIV networks for children outside London in the Children’s HIV National Network (CHINN) review of 2005. Each CHINN regional network links with one of the London Lead Centres, and each regional network has lead paediatrician(s) responsible for coordinating care in the region and linking to the London lead centre. Typically, clinical queries from any centre within the network are discussed with the tertiary centre, by either phone or email, usually with a single clinician's opinion given and with limited input from other specialists within the multidisciplinary team (MDT).

Virtual MDT clinics have been developed in adult HIV practice across the UK and USA for over a decade to support complex treatment decision making.^3–6^ These virtual MDT clinics typically meet to consider complex cases requiring antiretroviral decision making due to resistance, toxicity or the use of newer/off-label antiretroviral agents including those that require virtual clinic discussion as a condition of commissioning. Here, we report the experience of our paediatric virtual clinic (PVC) service between 2009 and 2013.

## Materials and methods

Retrospective database review of all patients referred to the PVC at the Family Clinic, St. Mary's Hospital, Imperial College Healthcare NHS Trust, London, between October 2009 and November 2013, was conducted. All referrals of children and adolescents aged 0–18 years were included. Young adults aged 18–21 years were included if they were transitioning from paediatric to adult services. In addition, prenatal referrals were included where advice involved neonatal management.

The PVC meets monthly and comprises four infectious disease paediatricians, two adult HIV physicians, an HIV virologist, paediatric HIV pharmacist, paediatric clinical nurse specialist and paediatric psychologist. Healthcare professionals are invited to submit anonymised patient data in a standardised form (see online supplementary appendix), including age at diagnosis, nadir/current CD4 count, viral load, opportunistic and coinfections, antiretroviral exposure, cumulative HIV-1 resistance mutations, drug side effects, adherence issues, HIV knowledge and disclosure status, neurocognitive functioning, social/quality of life issues and transition status. Each case is then presented to the PVC in a standardised format and recommendations are returned to the referring team by email and letter. Referrals are accepted from clinics within the network and from clinicians at other centres requesting advice. To assess PVC service outcomes, each referring centre was contacted by two of the team between September 2013 and January 2014 and information requested on clinical outcomes after PVC advice.

## Results

### Demographics

Between October 2009 and November 2013, 234 referrals were made for 182 children (median age 13 years, IQR 10–15 years). Of the 182 children referred, 82.4% (150/182) were discussed once, 13.2% (24/182) twice, 4.8% (7/182) three times and 0.5% (1/182) four times. Children were referred for more than one reason in 15 cases. Referrals came from 37 centres in 11 countries. Table 1 shows the referral patterns by year.

**Table 1:** Referral pattern to paediatric HIV virtual clinic by year 2009–2013 (N=234)

	Total referrals	2009*	2010	2011	2012	2013
Total	234	16	45	56	57	60
Paediatric virtual clinic centre (%)	112 (48)	9 (56)	26 (58)	26 (46)	29 (51)	22 (37)
Number of referrals in London (%)	24 (10)	1 (6)	4 (9)	7 (13)	5 (9)	7 (12)
Number of referrals in other UK centres (%)	83 (35)	6 (38)	15 (33)	20 (36)	20 (36)	22 (37)
Number of international referrals (%)	15 (6)	0	0	3 (5)	3 (5)	9 (15)

*Three-month data.

The majority of referrals were from our own centre (112/234, 48%), 10% (24/234) from other London centres and approximately one-third originated from other centres within the UK and Ireland. However, 2013 saw an increase in the number of international referrals mainly via the Paediatric European Network for the treatment of AIDS training network. Countries referring included Ukraine (n=4), Russia (n=4), Poland (n=1), Latvia (n=1), Cyprus (n=1), Australia (n=1), Malaysia (n=1) and New Zealand (n=2).

The most common referral reason was virological failure (44% (103/234 referrals in 80/182 children)), followed by regimen simplification (24% (57/234, 44/182)), complications of treatment (24% (56/234, 43/182)), restarting HAART after treatment interruption (7% (16/234, 10/182)) and starting HAART for the first time (3.4% (8/234, 8/182)). Reasons for referral are outlined in table 2.

**Table 2:** Reason for referral to paediatric HIV virtual clinic, episodes by year (N=234)

Referral reason	N=234	2009* (%) N=16	2010 (%) N=45	2011 (%) N=56	2012 (%) N=57	2013 (%) N=60
Control of viraemia						
Virological failure	103	11	24	20	23	26
Restart HAART	16	2	4	2	7	1
Start first line HAART	8	0	2	1	1	4
Simplification of regimen	57	3	10	19	14	11
Drug toxicity						
Dyslipidaemia	27	2	3	10	7	5
Abnormal liver function	11	0	1	3	3	4
Renal toxicity	9	1	1	1	3	3
Hypersensitivity	2	0	0	1	1	0
Neurological side effects	2	0	1	0	1	0
Other complications	5	0	2	1	1	1
Other						
Change to newer therapy	7	0	0	1	1	5
Thrombocytopenia	4	1	1	1	0	1
Poor immune reconstitution	3	1	1	0	0	1
Infant PEP	2	0	0	1	0	1
Coinfection HIV/TB	1	0	0	0	0	1
Other	1	0	0	0	0	1
Total	234	16	45	56	57	60

*Three-month data.

HAART, highly active antiretroviral therapy; PEP, postexposure prophylaxis for infant born to HIV-infected mother.

### Referrals for HAART advice due to viraemia

The majority of PVC referrals were for HAART advice due to increasing HIV viral load (44% (127/234 referrals in 98/182 children): referrals for failing HAART in 103 cases, 16 referrals in treatment-experienced children not currently on HAART and eight referrals to start HAART in treatment-naïve children.

#### Virological failure in treatment-experienced children

The majority of children were HAART experienced (90 children in 119 referrals). Sixteen children (11%, 10/90) had a period of treatment interruption before referral to clinic. In children who had previously received HAART, 82.6% (74/90) had HIV-1-associated drug resistance: single-class resistance 31.4% (23/74), dual class 37.2% (27/74), triple class 13.9% (9/74) and four class 1.2% (1/74). In total, 43 of 90 (48%) children achieved virological suppression after the first PVC referral, a further 8 of 14 (57%) following subsequent PVC discussion. Reasons for re-referral included ongoing adherence issues (6/14, 47%), social services involvement to enable families to engage in restarting treatment (3/14, 21%), toxicity from new regimen (2/14, 17%), clarification of regimen following viral tropism results (2/14, 17%) and one child resuppressed without change in medication.

#### Referrals for increasing viral load intreatment-naive children

Eight HAART-naïve children (8/98, 8%) were referred with increasing viral load, falling CD4 lymphocyte count and/or clinical disease consistent with criteria for HAART initiation. Seven had achieved virological suppression at the time of this study. One family required ongoing support from social services to engage with therapy.

### Referrals for HAART regimen simplification

A total of 24% (44/182) of children were referred to PVC for regimen simplification while on suppressive HAART, 40 of 44 (91%) with requests for a once daily regimen or lower pill burden in adolescents. Seven adolescents were referred on regimens that included newer HAART agents including twice daily raltegravir and darunavir/ritonavir. Forty-two children (42/44, 95%) remained virologically suppressed and adherent to treatment at follow-up. Two children require ongoing adherence support before switching HAART.

### Referrals for HAART advice due to drug toxicity or long-term HIV exposure

Forty-three children (24%, 43/182, 56/234) were referred with evidence of antiretroviral toxicity. Forty-eight per cent of referrals (21/43, 27/56) were for hypercholesterolaemia (serum cholesterol >5 mmol/L). A nucleoside-sparing regimen was recommended in 14% (3/21) of children. Additional dietetic input was recommended in 14% (3/21). Hypercholesterolaemia resolved in 42% (9/21). The PVC recommended the addition of a statin in one case. Four children did not change regimen as recommended by the PVC either because they or their parents/carers declined or because they moved away. Nine children with abnormal liver enzymes were referred in 11 referrals (11/56, 19.6%), three with hyperbilirubinaemia and cosmetically apparent jaundice secondary to boosted atazanavir that resolved with a change of protease inhibitor. Of the remaining eight children, hepatitis was associated with nevirapine in two cases. Liver enzymes returned to normal in all but one case on change of HAART. The remaining adolescent had evidence of non-alcoholic fatty liver disease ascribed to HAART exposure and excessive weight gain (body mass index 30).

Eight children (8/43 children in 9/56 referrals, 18%) were referred for proximal renal tubular leak associated with tenofovir. Renal tubular leak resolved in all but one case on cessation of tenofovir. This child was referred for additional renal investigations.

Four children on suppressive protease inhibitor-based HAART were referred with thrombocytopenia. In all cases, the recommendation was to start dapsone, reported to improve platelet numbers in adult case series and simplify to protease inhibitor monotherapy. Thrombocytopenia resolved in three cases.

Fourteen children referred for side effects of HAART/HIV were discussed at multiple PVCs. Reasons for re-referral were usually associated with ongoing drug toxicity despite changes to therapy and required ongoing discussion based on adult colleague experience of drug toxicity issues, dietician and psychological support.

### Referrals for HAART advice to avoid long-term drug side effects

Seven children were referred for consideration of a treatment switch to drugs with lower risk of long-term side effects: five to switch from didanosine and two switched from zidovudine to reduce risk of peripheral neuropathy/pancreatitis/non-cirrhotic portal hypertension and lipoatrophy, respectively.

### Recommendations for switch of HAART

In 178 of 234 (76%) referrals, HAART switching was recommended. Due to HIV-1-associated resistance mutations, the use of the off-license drug maraviroc was recommended in 17 of 178 (9.6%), raltegravir in 21 of 178 (11.8%) and etravirine in 1 of 178 (1%).

### Adherence support

Additional adherence support was recommended in 11.5% (21/182) children, including psychology, social services and Child and Adolescent Mental Health Service input, with gastrostomy insertion for HAART administration recommended in 10 of the 21 children (median age 12 years).

### Uptake of PVC advice

At the time of review, PVC advice had been followed in 80% (186/234) referrals, 74% in our centre (83/112), 80% (19/24) in other London centres, 88% (73/83) in other UK centres and 73% (11/15) internationally. Reasons for advice not being followed included two deaths in children in Eastern Europe before changes could be implemented, two UK children lost to follow-up despite social services involvement, additional re-referral for ongoing adherence and family support.

## Discussion

This retrospective study has demonstrated the diversity of referrals to our multidisciplinary virtual clinic for discussion of complex case management for children and adolescents living with HIV from a wide geographical region.

The majority of referrals in our cohort were for advice after virological failure with associated drug resistance in a treatment-experienced population. Pharmacokinetic and formulation issues exacerbate the problem of prescribing in children. As these children progress through paediatric services and into adult care, their life-long exposure to HIV and complex treatment histories make them a different population from their age-matched young adult counterparts. Data from the UK Collaborative HIV Paediatric Study cohort demonstrated that over one-third of children had experienced virological failure and were on second or subsequent line therapy at time of transition to adult care.^7^ In the paediatric setting, virological failure is most frequently due to poor adherence to HAART. In a large UK study, at the time of transition to adult care (median age 17.5 years), half of those who had ever taken HAART were triple-class experienced, and two-thirds had evidence of dual-class or triple-class resistance.^7^ Interpretation of resistance testing in this context can be complex, particularly in the presence of an expanding armoury of antiretroviral drugs and treatment decisions should be made with the support of pharmacists, clinicians and scientists with expertise in the field.^8^

Maintaining adherence is particularly difficult during adolescence, especially with a complex regimen if a once-daily, low pill burden option is not available due to the prior acquisition of resistance mutations.^9^ Non-adherence substantially increases the risk of disease progression, transmission of HIV to sexual partners/future offspring and the likelihood of further resistance to a particular drug or drug class. In this context, experienced psychologists within the PVC can offer expertise based on large case numbers that would be unfeasible in individual centres.

Family/caregivers are crucial to paediatric adherence because young children depend entirely on a caregiver to administer medications. Identifying someone responsible for medication administration is often problematic, especially when parents die or are impaired by complications of their own HIV infection. Responsibility for medication administration poses an evolving set of challenges in adolescents. The expectation that older children and adolescents should assume responsibility for taking their medication—in the setting of peer pressure to conform, chaotic social schedules and issues related to stigma and body image—is often unrealistic. Family members may have discrepant perceptions of a child's level of responsibility for medication, especially in families with older children.^10^ Specialist paediatric HIV-nursing input provides beneficial expertise in this setting within our PVC.

A significant proportion of PVC referrals surrounded complications of prolonged exposure to HAART. Protease inhibitors and nucleoside reverse transcriptase inhibitors exert their effects on fat distribution and lipid metabolism and are believed to act synergistically when used in combination.^11^ Fat redistribution syndrome is a complication of prolonged treatment and affects body image.^12–19^ Adults living with HIV are at increased risk of cardiovascular disease and there are also increasing data on dyslipidaemia in HIV-infected children.^15–17^ Switching to alternative antiretroviral agents has been recently associated with a reduction in cholesterol in paediatric populations, mirroring data seen earlier in adult cohorts.^20^ In addition, changes in body fat composition may adversely affect treatment adherence posing further conundrums for paediatricians and can be discussed in a PVC setting with input from adult and paediatric healthcare professionals.

Our study has limitations. First, data were collected retrospectively, thus we were unable to gather demographic and social information not entered on the referral form. However, the majority of children are well known to their local service and have a long relationship with their clinicians, so we were able to ascertain the majority of pertinent information by contacting the local team. In addition, we relied on the referring clinicians reporting of HAART exposure, current and archived resistance mutations hence our reporting of resistance in this cohort must be taken as a minimum. Finally, reporting of drug side effects and resolution relied on clinician reporting. Therefore, we have not been able to accurately ascertain all potential side effects from exposure to HAART in this patient group.

There is limited information available to paediatricians and primary care physicians about most rare childhood diseases, yet physicians need access to evidenced-based information for themselves and their patients to optimise care.^21^ National and international programmes advocate the use of clinical networks for rare diseases, to share information and to optimise and standardise management and education.^22^ Virtual communities are emerging to provide patient and clinician information on chronic diseases in Europe^23^ ^24^ and secure internet is widely used in paediatric services such as diabetes care to improve education and management.^25^ A model of care such as the PVC provides added benefits over and above the traditional MDT because it is not bound by geography. Team membership can be flexible and allows specialists working at different locations to discuss rare or difficult cases and provides opportunities for professional development as members of the team may interact with specialists, such as adult physicians, with experience outside of their immediate working environment. In terms of infrastructure, the virtual MDT has the benefit of flexible location that can be modified to the needs of the specialists involved. A standard information technology platform could be used by multiple teams at multiple locations, thus enabling transparency of the discussion and with the future potential of allowing patient participation in an on-line discussion of their care. The virtual clinic for HIV goes some way to meeting the goals set out by the European Organisation for Rare Diseases and CHIVA to improve and standardise care, education and decision making and could provide a care model for other complex, rare and chronic conditions best met by experienced multidisciplinary input.

## Conclusions

The PVC is a valuable tool to address some of the limitations and difficulties associated with the management of complex chronic diseases that are rare in individual practice within an NHS network. The use of the virtual clinic allows access to a diverse range of expertise irrespective of location and enables the best opportunity for providing expert care in complex or unusual problems, especially as the child enters adolescence.

## Supplementary Material

Web supplement
