# Supplementary material for: Virtual support for paediatric HIV treatment decision making
Source: Arch Dis Child. 2014 Dec 30;100(6):527–31. doi: 10.1136/archdischild-2014-307019 (PMC4453589; doi:10.1136/archdischild-2014-307019)
Supplement: Web supplement [file archdischild-2014-307019-s1.pdf]

Supplementary Table – regimen referred for simplification

**Regimen**	**N (%)**
Kaletra + 2NRTI	23 (46)
Kaletra + 3NRTI	12 (24)
Kivexa + boosted PI	3 (6)
Kivexa, nevirapine	2 (4)
Kivexa, tenofovir, darunavir/ritonavir, raltegravir	1 (2)
Kivexa, tenofovir, etravirine, raltegravir	1 (2)
Truvada, darunavir/ritonavir, raltegravir	3 (6)
Zidovudine, darunavir/ritonavir, raltegravir	2 (4)
Other regimen containing darunavir/ritonavir and NRTI	3 (6)
**TOTAL**	**50**

Kaletra: lopinavir/ritonavir; NRTI – nucleoside reverse-transcriptase inhibitor; Kivexa – abacavir/lamivudine; PI – protease inhibitor; Truvada – embricitabine/tenofovir
